# Deep Learning Techniques with Genomic Data in Cancer Prognosis: A Comprehensive Review of the 2021–2023 Literature

**DOI:** 10.3390/biology12070893

**Published:** 2023-06-21

**Authors:** Minhyeok Lee

**Affiliations:** School of Electrical and Electronics Engineering, Chung-Ang University, Seoul 06974, Republic of Korea; mlee@cau.ac.kr

**Keywords:** deep learning, cancer prognosis, survival analysis, genomic data, biomedical data analysis

## Abstract

**Simple Summary:**

The ongoing advancements in deep learning, notably its use in predicting cancer survival through genomic data analysis, calls for an up-to-date review. This paper inspects notable works from 2021 to 2023, underlining essential developments and their implications in the field. We offer a comprehensive review of the research, selective paper choice, and thorough analysis of prevailing trends, contributing to a better understanding of deep learning’s potential in this vital domain.

**Abstract:**

Deep learning has brought about a significant transformation in machine learning, leading to an array of novel methodologies and consequently broadening its influence. The application of deep learning in various sectors, especially biomedical data analysis, has initiated a period filled with noteworthy scientific developments. This trend has majorly influenced cancer prognosis, where the interpretation of genomic data for survival analysis has become a central research focus. The capacity of deep learning to decode intricate patterns embedded within high-dimensional genomic data has provoked a paradigm shift in our understanding of cancer survival. Given the swift progression in this field, there is an urgent need for a comprehensive review that focuses on the most influential studies from 2021 to 2023. This review, through its careful selection and thorough exploration of dominant trends and methodologies, strives to fulfill this need. The paper aims to enhance our existing understanding of applications of deep learning in cancer survival analysis, while also highlighting promising directions for future research. This paper undertakes aims to enrich our existing grasp of the application of deep learning in cancer survival analysis, while concurrently shedding light on promising directions for future research in this vibrant and rapidly proliferating field.

## 1. Introduction

Deep learning has prompted a notable shift in the machine learning field, introducing a new period of innovation and discovery [1,2,3]. This shift has led to the creation of various new methodologies, such as image generation [4,5,6] and natural language processing [7,8,9,10]. These enhanced techniques have expanded the applicability of deep learning, enabling its incorporation into various fields, including biomedical data analysis [11,12,13], engineering design [14,15], and computer vision [16,17,18,19].

Cancer prognosis using genomic data is a complex and multifaceted field. The intricate nature and vastness of genomic data poses significant challenges to conventional computational techniques. Traditional methods often struggle with the high dimensionality of this data and the need for manual feature selection, which might not capture the underlying biological complexities. This has led to a pressing need for more advanced techniques capable of extracting meaningful insights from such intricate datasets.

Deep learning, a subset of machine learning, has emerged as a potent tool in handling and interpreting complex genomic data. By learning hierarchical representations directly from data, it can handle the high-dimensionality of genomic datasets, eliminating the need for manual feature selection and potentially capturing more relevant biological signals. Advancements of deep learning have substantially influenced many scientific domains [20]. In particular, deep learning has made a strong impact on cancer prognosis, where genomic data interpretation for survival analysis has become a central area of research [21,22]. The ongoing developments of deep learning methodologies and their ability to extract complex patterns from high-dimensional genomic data have considerably improved our understanding of cancer survival [23,24,25], highlighting the need for a detailed review of this rapidly evolving field.

The present review is a carefully curated exploration of the most recent and impactful studies in the field, spanning a period of three years, from 2021 to 2023. This tight temporal focus represents the dynamism and swift pace of development within the field of deep learning, requiring a current review of the most recent advancements. The unique contribution of this review lies in its wide-ranging coverage of the recent research, its rigorous paper selection process, and its thorough analysis of the main trends and methodologies in the field.

The architecture of this review paper is thoughtfully designed to offer a comprehensive explanation of the growing domain of deep learning applied to cancer survival analysis grounded in genomic data. This paper begins with the aim of the review discussed in Section 2 as well as a detailed investigation presented in Section 3. This initial stage meticulously outlines the fundamental principles guiding the selection of the research articles, provides clarity on the criteria leading to the omission of certain papers, examines the range of journals hosting these research endeavors, and conducts a quantitative analysis of the chosen papers, primarily focusing on citation frequency and years of publication.

Following this initial groundwork, we explore a diverse array of methodologies that find their application in survival analysis, each accorded its own dedicated section for an in-depth discussion. These include Section 4, dedicated to recent studies with autoencoders, Section 5, discussing ensemble methods and models, Section 6, focusing on transfer learning, Section 7, dealing with multi-modal and multi-omics frameworks, Section 8, explicating manifold representation learning, and Section 9, elucidating standard deep learning.

Finally, we encapsulate our findings and insights in Section 10. Here, we distill the essence of our investigation, emphasizing the important discoveries while spotlighting promising trajectories for future research. This manuscript presents a comprehensive review of recent advancements in deep learning techniques applied to genomic data for predicting cancer prognosis. The contributions of this work are fourfold:

- Thorough review of deep learning techniques applied to genomic data in cancer prognosis;
- Focus on the most recent literature from 2021 to 2023, providing readers with an up-to-date understanding of this rapidly evolving field;
- Categorization of the deep learning methodologies used in cancer prognosis prediction using genomic data;
- Inclusion of only those studies that intersect the domains of cancer, prognosis (survival analysis), deep learning, and genomic data.

## 2. Preliminaries

### 2.1. Cancer Survival Analysis

Cancer survival analysis, often encapsulated under the broader umbrella of survival analysis [24,26,27], is a branch of statistics dealing with death (or failure) in biological organisms and failure in mechanical systems. It focuses on the expected duration of time until an event of interest (such as death, recurrence of disease, or another adverse event) occurs.

Traditionally, survival analysis techniques involve evaluating and interpreting time-to-event data [28], where the event is commonly death, or in the case of cancer survival analysis, the death caused specifically by cancer. This includes understanding the risk factors associated with the time-to-event outcome and predicting the survival function or the time to event for a given individual.

The survival function, S(t), which gives the probability that a person survives longer than some specified time, *t*, and the hazard function, h(t), that describes the risk of death at time *t* conditional on survival until time *t*, are the key functions in survival analysis [29]. The *survival function*, S(t), is defined as the probability that the time to event is greater than some time *t*, i.e., the probability of surviving past time *t*.

(1) S(t)=P(T>t)

where *T* is the time to event.

The *hazard function*, h(t), on the other hand, is the event rate at time *t* conditional on survival until time *t* or later (i.e., T≥t). It can be considered as the risk or the force of mortality, or failure, at a particular instant.

(2) $$h\left(t\right)=\lim_{\Delta t\to 0}\frac{P(t\leq T<t+\Delta t|T\geq t)}{\Delta t}$$

These functions form the core of survival analysis, allowing researchers to describe and summarize survival data, identify important factors, and create models for predicting future outcomes. In cancer survival analysis, researchers aim to analyze and interpret data related to cancer patients’ survival time, considering various features such as patient’s age, type and stage of cancer, treatment methods, and genetic factors, among others. This helps in understanding the effectiveness of different treatment options and the prognosis of the disease and can aid doctors and patients in making informed decisions related to treatment strategies. Hence, cancer survival analysis is crucial in cancer research. The insights gained from these analyses help in understanding the disease better, identifying factors that influence survival rates, and ultimately improving treatments and increasing survival rates.

### 2.2. Artificial Intelligence, Machine Learning, and Deep Learning

Artificial intelligence (AI), machine learning, and deep learning are often used interchangeably in various contexts. However, they are inherently different, representing successive layers of abstraction and complexity [30].

AI is a broad field that encompasses the concept of creating intelligent machines capable of simulating human intelligence, including problem-solving, learning, planning, and understanding language. AI has been a subject of study since the inception of computer science and can be divided into two main types: narrow AI, which is designed to perform a narrow task, and general AI, which can perform any intellectual task that a human being can.

Machine learning, a subset of AI, refers to the design and development of algorithms that allow computers to learn from and make decisions or predictions based on data. These algorithms operate by building a mathematical model based on input data (i.e., ’training data’) to make predictions or decisions without being explicitly programmed to perform the task.

Deep learning, a further subset of ML, constitutes a collection of algorithms that employs a hierarchical level of artificial neural networks to carry out the process of machine learning. The hierarchy of artificial neural networks mimics the human brain in a nuanced manner while providing a profound improvement on traditional machine learning algorithms. By using a cascading structure of multiple layers for feature extraction and transformation, each successive layer uses the output from the previous layer as an input, thus enabling the algorithm to learn from data in a progressive and hierarchical manner.

Indeed, the concept of deep learning takes inspiration from the functioning of the human brain, more specifically, the neural activations. In a similar vein, artificial neurons within a deep learning network are triggered into an ’active’ state when the sum of their received signals surpasses a predefined threshold. This paradigm elegantly echoes the biological process, whereby a neuron fires an action potential when a certain threshold of input stimulation is reached. Further translating this analogy into the mathematical domain, deep learning operationalizes this neural activation concept through the means of matrix multiplication. In a structured manner, each artificial neuron’s inputs are multiplied by their associated weights. These products are then accumulated, and if the cumulative sum exceeds the neuron’s specific activation threshold, the neuron is activated, transmitting its signal to the next layer in the network. This process, carried out across thousands or even millions of neurons and multiple layers, forms the mathematical underpinning of the deep learning paradigm, thereby facilitating a robust and nuanced modeling of complex data patterns.

### 2.3. Deep Learning Techniques in Cancer Prognosis with Genomic Data

Deep learning techniques have emerged as powerful tools for dealing with complex and high-dimensional data, such as genomic data in cancer prognosis. Genomic data provides a wealth of information that can be used to predict patient outcomes and tailor treatments to individual patients. Deep learning methods, given their capacity to model complex, non-linear relationships and their ability to handle large amounts of high-dimensional data, are particularly suited to this task.

**Figure 1:**
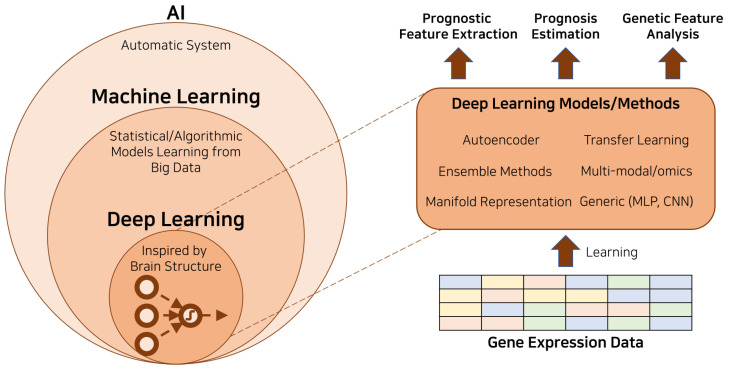
Illustrative diagram depicting the application of deep learning in cancer prognosis using genetic data.

The deep learning techniques typically used in cancer prognosis with genomic data include but are not limited to multi-layer perceptron (MLP), convolutional neural networks (CNNs), and autoencoders. These methods have proven effective in a variety of tasks, including predicting patient survival times, classifying different cancer types, and identifying genetic markers for specific cancers. In this review, we will provide an overview of these methods and discuss their application in the field of cancer prognosis with genomic data. Figure 1 depicts an illustrative diagram that portrays the application of deep learning in cancer prognosis utilizing genetic data. Table 1 provides a comprehensive overview of the recent studies, effectively organizing them into distinct categories.

## 3. Literature Review and Paper Selection

### 3.1. Principles Underpinning the Selection of Research Articles

This review specifically focuses on the intersection of four primary research areas: (1) cancer, (2) prognosis (survival analysis), (3) deep learning, and (4) genomic data. The studies included in this review satisfy all four of these conditions. To ensure a comprehensive and relevant review, our paper selection was guided by a strict set of principles and employed a meticulous strategy.

The primary source of research articles was the scholarly search engine Web of Science (WOS), using a set of keywords around the themes of deep learning, survival analysis, and genomic data. We focused on articles published in peer-reviewed journals between 2021 and 2023, excluding review articles, perspectives, and similar types of literature. The selection of articles from peer-reviewed journals aimed to uphold the quality of our review by only including scientifically sound, thoroughly vetted, and impactful studies. Additionally, the selected papers were categorized based on the specific deep learning methods employed, providing a structured overview of various methodologies in the field.

### 3.2. Exclusion Criteria

Our selection process involved a rigorous curation and a manual scrutiny phase to align with the scope and objectives of our review. The key exclusion criteria were as follows:

- *Data type*: Articles primarily using image data were excluded, with exceptions for those incorporating both image data and genomic data in a multi-modal deep learning approach;
- *Research objectives*: Papers focusing solely on cancer subtype prediction without survival analysis were omitted;
- *Analytical methods*: Articles using only conventional machine learning methods were excluded, with exceptions for those using both machine learning and deep learning.

This curation process resulted in a selection of 79 papers, providing a focused insight into the current state of deep learning for cancer survival analysis with genomic data.

### 3.3. Publication Platforms and Analysis of Selected Papers

**Figure 2:**
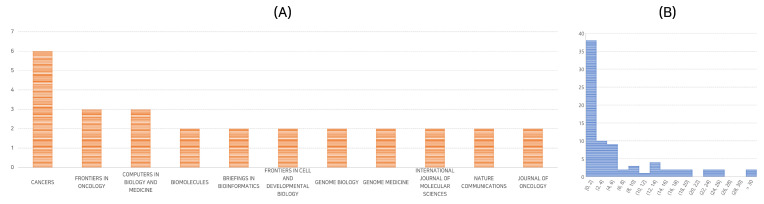
Quantitative analysis of the publications: (**A**) Distribution of papers across journals; (**B**) Distribution of citation frequency. The *x*-axis denotes the number of citations, while the *y*-axis represents the number of publications.

Our selected papers were published across a diverse range of academic journals, indicating the interdisciplinary interest in deep learning for cancer survival analysis using genomic data. The most frequent publication platform was “Cancers”, hosting six of the seventy-nine selected papers, a mere 8% of the total. Other common platforms included “Frontiers in Oncology” and “Computers in Biology and Medicine”, each with three papers. A detailed distribution of papers across different journals is shown in Figure 2A.

We further investigated the selected papers in terms of their citation frequency and publication years. The median number of citations for the selected papers is 3, with the mean at 6.8, suggesting a few highly-cited papers. In terms of publication years, 2021 and 2022 were particularly active, with 33 and 39 papers published, respectively. As of April 2023, 7 papers have already been published. Figure 2B provides a more detailed overview of the citation frequency.

## 4. Survival Analysis with Autoencoders

Autoencoders [110], a specific type of artificial neural network, have emerged as a central tool in bioinformatics, particularly in the context of cancer prognosis and subtype prediction. Autoencoders are unsupervised learning algorithms that aim to identify low-dimensional features, or encodings, of high-dimensional data. In the genomic context, these encodings often represent important biological characteristics that are crucial in understanding the underlying processes of cancer development and progression.

The structure of an autoencoder is composed of an encoder and a decoder, functioning in unison to create a compressed representation of the input data. This compressed representation, often termed as the bottleneck or latent space, captures the essential features of the input data. By optimizing the network to reconstruct the input data from this compact representation, the autoencoder learns to filter noise and preserve the most informative features, making it particularly useful for complex, high-dimensional biological data.

Recent trends in bioinformatics have seen a surge in the use of autoencoders, particularly deep autoencoders, to analyze multi-omics data for cancer prognosis and subtype prediction. Multi-omics data refers to the integration of genomic, transcriptomic, proteomic, and other types of biological data to provide a comprehensive understanding of biological systems. The high dimensionality and noise in multi-omics data pose significant challenges for traditional machine learning methods, necessitating the development of robust deep learning approaches. Autoencoders, with their capacity for unsupervised learning and dimensionality reduction, have proven to be an effective solution.

A common approach in these studies involves leveraging the autoencoder for feature extraction from high-dimensional omics data, followed by survival analysis or clustering for cancer prognosis and subtype prediction. The extracted features often reveal meaningful biological insights, such as identifying differentially expressed genes or survival-related features, and can be further utilized for downstream analysis or validation. Moreover, autoencoders have been extended to integrate multi-omics data, facilitating a holistic understanding of cancer biology and improving the predictive performance of survival models. Table 2 summarizes these recent studies.

The application of deep learning techniques, particularly autoencoders, to genomic data has seen a rise in popularity due to its effectiveness in cancer prognosis. Chai et al. [31] devised an innovative approach that applied a denoising autoencoder to integrate multi-omics data. This approach, when tested on 15 different cancer types from the TCGA database, exhibited an average improvement in C-index values of 6.5% over traditional methods. Notably, the model continued to demonstrate effective prediction capabilities with a C-index of 0.627, even when solely utilizing mRNA data.

In a similar vein, Kirtania et al. [32] developed a unique deep learning strategy, DeepSGP, which harnessed the power of autoencoders to predict the survival of glioblastoma multiforme (GBM) patients. By using RNA-seq data from 129 GBM samples, the authors leveraged EdgeR for DEGs selection, and then reduced these through correlation-based analysis. Autoencoders, among other feature subset selection and extraction algorithms, were subsequently employed, with DeepSGP demonstrating superior performance by achieving an impressive accuracy of 0.83 and an AUC of 0.90.

Several studies have focused on comparing the performance of autoencoders to other data fusion techniques. Franco et al. [33], for instance, compared four different autoencoders for cancer subtype detection using multi-omics data. This comparison showed that autoencoders were generally more successful than standard data fusion techniques such as principal component analysis (PCA), kernel PCA, and sparse PCA. The study underscored the potential of autoencoders in detecting significant differences in survival profiles, hence facilitating accurate patient subgroup prediction.

Expanding the application of autoencoders, Song et al. [34] used an autoencoder-based model to predict prognosis in colorectal cancer. By integrating DNA methylation, RNA-seq, and miRNA-seq data, the study was able to identify survival-related features, demonstrating that the autoencoder-based strategy outperformed other transformation strategies. Similarly, Lai et al. [35] presented a disease network-based deep learning approach that combined genomic data and an autoencoder model to characterize melanoma. This approach led to the identification of three distinct patient subtypes with varying survival times.

Moving on to another variant of autoencoder application, Yang et al. [36] introduced deep subspace fusion clustering (DSFC), an extension of similarity network fusion. This technique, which leveraged an auto-encoder and data self-expressiveness approaches, was used to integrate multi-omic data for cancer subtype prediction. DSFC showed promising results, demonstrating comparable or even better performance than many current integrative methods when evaluated across six different cancer types.

Continuing in this line of study, Zhang et al. [37] implemented a deep learning framework based on autoencoders to integrate multi-omics data for muscle-invasive bladder cancer (MIBC), identifying two distinct subtypes with notable differences in overall survival. The study also identified a potential biomarker, KRT7, for MIBC risk, affirming the robustness of their model across various validation datasets. Similarly, Tian et al. [38] and Zhang et al. [39] both utilized an autoencoder-based approach to identify survival-sensitive subtypes of gliomas and to propose a deep learning model integrating multi-omics data for disease subtype identification, respectively.

Transitioning from traditional autoencoders, Al Mamun et al. [40] proposed a deep learning algorithm, the concrete autoencoder (CAE), to identify prognostic long non-coding RNAs (lncRNAs) for 12 different types of cancers. The authors proposed a variant of the original algorithm, a multi-run CAE (mrCAE), to counter the stochastic nature of CAE and identify a more stable set of features. The resultant model was found to outperform the single-run CAE and other feature selection techniques, achieving an accuracy of 95%.

Madhumita and Paul [41] proposed an autoencoder-assisted cancer subtyping framework that uses a sparse autoencoder neural network to capture the latent space of multi-omics data. The authors demonstrated the efficiency of this framework across five different multi-omics cancer datasets from TCGA. The framework provided a robust information bottleneck by selecting a compression of 10–20% of input features with L1 regularization penalty, thereby enabling the prediction of clinically significant cancer subtypes. The study highlights the potential of autoencoder-assisted multi-omics integration in predicting cancer subtypes and provides insights into the biological and clinical implications of the identified subtypes.

## 5. Survival Analysis with Ensemble Deep Learning Methods and Models

The ensemble of deep learning models constitutes a widely employed paradigm in bioinformatics, geared towards improving the predictive performance of individual models [111]. This approach harnesses the collective intelligence of multiple learning algorithms to obtain superior predictive power, often leading to improved accuracy and robustness of the model. The ensemble methodology typically involves training multiple models, each with different architectures or hyperparameters, and combining their outputs to yield a final prediction. This combination can be accomplished through a variety of methods, including simple averaging, weighted averaging, or more sophisticated approaches such as stacking, bagging, and boosting.

Emerging trends in the field of bioinformatics have embraced the ensemble methodology, integrating it with deep learning models to tackle complex problems such as cancer prognosis prediction, gene identification, and biomarker classification. This trend stems from the unique challenges presented by the vast, high-dimensional, and often noisy biological data, which necessitate robust and versatile models. The ensemble of deep learning models offers the advantage of capturing different facets of the data through individual models, thereby providing a more comprehensive representation of the underlying biological processes.

One common strategy involves the fusion of deep learning algorithms with traditional machine learning methods or statistical models, such as survival analysis, random forest classifiers, or regression models. This combination leverages the strengths of both paradigms, harnessing the capacity of deep learning for feature extraction and representation learning, and the interpretability and statistical rigor of traditional models. Moreover, advanced techniques such as fuzzy logic systems, Bayesian optimization, and generative adversarial networks have been integrated into the ensemble framework, further enhancing the model’s capabilities.

The use of ensemble deep learning techniques to improve the prognosis of cancer patients has seen notable developments in the past few years (Table 3). For instance, Poirion et al. [42] established DeepProg, a robust prognostic prediction model comprising an amalgamation of machine learning and deep learning models. DeepProg was assessed on liver and breast cancer datasets, where it showcased superior predictive performance, with C-index values ranging from 0.68 to 0.80. Notably, the model’s pan-cancer analysis associated poor survival subtypes with a range of biological processes, including extracellular matrix remodeling, immune deregulation, and mitosis.

Along a similar vein, Arya and Saha [44] introduced a two-phase model that merges random forest classifiers with gated attentive deep learning models, aiming to enhance the prognosis prediction in breast cancer patients. This innovative approach demonstrated a considerable improvement in survival estimation for breast cancer patients when benchmarked against conventional methods. Further building on the theme of ensemble learning, Kaur et al. [46] proposed a parallel Bayesian hyperparameter optimized Stacked ensemble (BSense) model for predicting breast cancer survival. This amalgamation of deep neural networks (DNNs), gradient boosting machines, and distributed random forests (DRFs) was further enhanced by applying Bayesian optimization with Gaussian processes for hyperparameter tuning. On evaluation using several datasets, including TCGA, METABRIC, Metabolomics, and RNA-seq datasets, the model exhibited superior performance in predicting breast cancer survival.

Several research efforts have proposed unique integrative models by combining deep learning algorithms with other techniques. For instance, Yang et al. [43] proposed FuzzyDeepCoxPH, an innovative model that blends deep learning algorithms, a fuzzy logic system, and conventional survival analysis. This model aimed to identify high-risk missense mutation variants and genes strongly associated with cancer mortality. By integrating these diverse techniques, the model effectively provides a comprehensive estimation of cancer mortality risk, highlighting its efficacy in distinguishing high-risk variants and genes related to cancer mortality.

Another novel approach was undertaken by Zi et al. [45], who developed a deep neural network model for predicting survival risk in patients with hepatocellular carcinoma (HCC). This model applied various machine learning algorithms such as K-Means, random forests, and LASSO regression to identify new HCC subtypes and select key genes, demonstrating high accuracy exceeding 93.3%.

Recent advancements have also been seen in the use of generative adversarial networks (GANs) and self-supervised learning. Tamilmani et al. [47] developed a model that combines a deep convolutional GAN and modified convolutional neural network optimized with a mayfly optimization algorithm. This approach aims to balance the dataset and improve the classification performance for cancer miRNA biomarker classification. In contrast, Chen and Wei [48] presented a survival prediction model that combines a deep survival forest and self-supervised learning for adaptive learning of high-dimensional genomic data. This model demonstrated superior performance in terms of the C-index and brier score when compared with advanced survival analysis methods, suggesting its potential utility in personalized treatment decision-making.

The use of deep learning techniques in predicting overall survival in cancer patients has been explored by Carreras et al. [49]. They employed an algorithm that integrates a multilayer perceptron artificial neural network, radial basis function, GSEA, and conventional statistics. This integrated model facilitated dimensionality reduction by correlating 20,862 genes with 28 MCL prognostic genes. This approach resulted in the identification of 58 genes that predict survival with high accuracy, demonstrating its potential in predicting the survival of a large pan-cancer series, including common cancers such as lung, breast, colorectal, prostate, stomach, and liver.

## 6. Survival Analysis with Transfer Learning

Transfer learning in the context of deep learning is a technique where a pre-trained model is adapted for a different but related problem [112]. This approach stems from the observation that the features learned by deep learning models on a specific task can serve as a useful starting point for learning on other tasks. This is particularly true in domains where labeled data is limited or expensive to obtain, as is the case with many problems in the biomedical field.

The primary motivation behind transfer learning is to leverage the knowledge gained from training on large-scale datasets (such as ImageNet for image classification tasks) and apply it to tasks where the amount of available labeled data might be scarce. For instance, a model pre-trained on a large image dataset might have learned useful low-level features, such as edge or color detectors, that can be applied to a medical imaging task.

Transfer learning techniques have been gaining popularity in recent years, with an increasing trend towards their application in various fields, such as computer vision, natural language processing, and bioinformatics. One of the most common approaches in transfer learning involves fine-tuning, where a pre-trained model is trained further on a target task, usually with a smaller learning rate, to adapt its parameters to the specifics of the new task.

The use of transfer learning in deep learning techniques for cancer prognosis has seen a surge of interest in recent years, as summarized in Table 4. An excellent example of this is the study conducted by Chai et al. [77], where they developed a transfer learning-based Cox proportional hazards network (TCAP), designed to integrate multi-omics data for bladder cancer prognosis predictions. This model outperformed its predecessors, achieving a C-index of 0.665. Additionally, they trained an XGBoost model exclusively on mRNA data, which demonstrated a commendable C-index of 0.621 on the TCGA dataset and an average C-index of 0.637 on three other datasets.

Parallel to this, Johnson et al. [78] proposed a unique deep transfer learning framework called diagnostic evidence gauge of single cells, which transfers disease information from patients to individual cells. This model, applied to single-cell and patient bulk tissue transcriptomic datasets from a range of diseases, such as glioblastoma multiforme and Alzheimer’s disease, showed encouraging results.

Focusing on the tumor microenvironment, Shi et al. [79] employed a deep learning model for colorectal cancer prognosis prediction based on the histopathologic features. Utilizing the VGG19 architecture and a transfer learning strategy, the researchers automated the quantification of the tumor microenvironment in whole slide images. This model achieved an impressive accuracy of 94.2% in recognizing different tissue types. The tumor microenvironment signature, developed from the extracted features, demonstrated a significant association with progression-free survival, emphasizing the importance of incorporating histopathological features in prognosis prediction.

Expanding the scope of current deep learning approaches, Meng et al. [80] presented SAVAE-Cox, a novel framework that combines an attention mechanism with an adversarial transfer learning strategy. This unique approach was used for survival analysis of high-dimensional transcriptome data. The model was trained on 16 types of TCGA cancer RNA-seq datasets, and it showed superior performance over other state-of-the-art survival analysis models, such as Cox proportional hazard model, Cox-lasso, Cox-ridge, Cox-nnet, and VAECox, in terms of the concordance index.

## 7. Survival Analysis with Multi-Modal and Multi-Omics Frameworks

Multi-modal deep learning and multi-omics methods are swiftly gaining recognition in the biomedical domain, particularly in the study of cancer prognosis and diagnosis [113,114]. Multi-modal deep learning refers to the combination of various types of data, such as images, text, or structured data, in a single model, which can lead to more comprehensive and accurate predictions. In the context of cancer research, multi-modal models often integrate data from different imaging techniques, such as CT and MRI scans, along with histopathological images, genomic data, and clinical information. This approach allows for the incorporation of heterogeneous information into the prediction models, thereby increasing their interpretability and performance.

Multi-omics methods, on the other hand, provide an integrative view of the molecular landscape of cancer by combining multiple omics layers, including genomics, transcriptomics, proteomics, metabolomics, and epigenomics. This comprehensive view allows for the unraveling of the complex interplay of different biological processes and pathways involved in cancer. The integration of multi-omics data can be achieved through various machine learning and deep learning techniques, including tensor decomposition, variational autoencoders, and deep belief networks.

Recent trends in cancer research point towards a convergence of multi-modal deep learning and multi-omics methods, offering a more holistic view of cancer biology. This approach leverages the power of deep learning to extract meaningful patterns from high-dimensional and heterogeneous data, leading to improved prediction performance and better biological insights. Techniques such as gating-based attention mechanisms, convolutional neural networks, graph convolutional networks, and deep tensor survival models are being employed to integrate multi-modal and multi-omics data.

Recent research in the field of oncology has seen a convergence of efforts to integrate multi-modal and multi-omics data for comprehensive cancer prognosis, as shown in Table 5. A number of such studies utilize deep learning models and focus on specific types of cancer, revealing noteworthy methodological insights and advancements. Schulz et al. [50] and Chen et al. [51] both developed multimodal deep learning models for prognosis prediction, but with distinct emphases. Schulz et al. [50] trained their model on a combination of multiscale histopathological images and genomic data, with the model demonstrating significant prognostic value. Chen et al. [51], however, proposed a fusion strategy for histopathology and genomic features, enhancing the control of representational expressiveness via a gating-based attention mechanism.

Efforts to integrate multi-omics data have been abundant in the literature, providing predictive survival models for various cancers. Zhang et al. [52] proposed a deep tensor survival model integrating multi-omics data, demonstrating improved prediction performance over models using individual genomic data. Similarly, Malik et al. [53] and Hassanzadeh et al. [54] utilized neural network frameworks to integrate multi-omics data, achieving high predictive accuracy for survival and drug response.

In terms of methodological versatility, the studies by Zhang et al. [55] and Wei et al. [56] stand out. OmiEmbed [55] is a unified multi-task deep learning framework designed for multi-omics data, demonstrating superior performance across a variety of tasks, including dimensionality reduction and survival prediction. Wei et al. [56] applied their deep learning approach to a cohort of 417 prostate cancer patients, identifying relapse-associated subgroups and highlighting the potential for early intervention strategies.

Some studies have innovated by combining radiomic features with genomic data for survival analysis. For instance, Chen et al. [60] integrated these data types to improve survival prediction for non-small-cell lung cancer patients. In a similar vein, Islam et al. [63] synthesized missing MRI modalities to integrate gene expression data with radiomic features, thus enhancing overall survival prediction.

Notably, a significant number of these studies underscored the importance of integrating multi-modal and multi-omics data for understanding cancer characterization and improving prediction accuracy [57,58,59,61,62]. Models such as the multi-prognosis estimation network developed by Choi and Lee [61] introduced gene attention layers for each data type, enabling the identification of prognostic genes. Others, such as MultiCoFusion [69], demonstrated the value of multi-task learning in improving performance across tasks in both single-modal and multi-modal data.

## 8. Survival Analysis with Manifold Representation Learning

Manifold representation learning with deep learning is an area of research focused on learning the underlying structure or geometry of the high-dimensional data [115]. This approach seeks to uncover a low-dimensional representation (a manifold) that captures the essence of the data, thereby making it more interpretable, manageable, and amenable to subsequent analysis. Manifold learning techniques, when combined with deep learning, can leverage the ability of neural networks to learn complex, non-linear transformations, enabling them to discover intricate manifold structures in high-dimensional spaces.

Deep learning-based manifold representation learning has seen significant development in the past few years, driven by the increasing availability of large and complex datasets, especially in domains such as bioinformatics, computer vision, and natural language processing. A common theme among these developments is the integration of manifold learning techniques with deep learning architectures, aiming to improve the efficiency, interpretability, and performance of the models.

Manifold learning techniques often rely on the assumption that high-dimensional data, such as images or gene expression profiles, inherently reside on a low-dimensional manifold embedded within the high-dimensional space. The goal is to discover this manifold and use it as a new, more compact, and potentially more informative representation of the data. This can be particularly valuable in bioinformatics, where data are often high-dimensional and complex, making traditional analysis methods challenging.

Several studies have addressed the development of deep learning methods for cancer prognosis using manifold representation learning, reflecting the diverse nature of cancers and their treatments (Table 6). Among these, Zhang and Kiryu [81] introduced an unsupervised clustering methodology, MODEC, leveraging manifold optimization and DL techniques to integrate multi-omics data for identifying cancer subtypes. MODEC’s utility was demonstrated through its successful application on the cancer genome atlas (TCGA) datasets, emphasizing its effectiveness in distinguishing clinically significant cancer subtypes. Conversely, Li et al. [85,86] devised DL models targeting specific types of cancer, such as colorectal and oral squamous cell carcinoma, respectively. Both studies’ models were designed to predict survival outcomes, with the former model also assessing the benefits of adjuvant chemotherapy.

In a different line of research, Zhang et al. [82] and Gupta et al. [83] exploited deep learning to design tools to handle high-dimensional datasets effectively. Zhang et al. established the deep Bayesian perturbation Cox network (DBP) to predict survival outcomes in cancer patients, notably proficient when working with large high-dimensional datasets with a substantial portion of censored samples. Gupta et al. introduced the continuous representation of codon switches (CRCS), a deep learning-based method producing numerical vector representations of mutations, which was applied to detect cancer-associated somatic mutations, identify driver genes, and predict patient survival.

Several investigations focused on the exploration of specific factors and their influences on cancer prognosis. Kim et al. [84] utilized deep learning to investigate tumor-infiltrating lymphocytes, signifying their potential in the tumor microenvironment. Using CIBERSORT, the researchers designed a deep learning-based model for predicting survival in oral cancer. Skead et al. [88] applied deep learning to study age-related clonal hematopoiesis (ARCH), a condition linked with an elevated risk of blood malignancies. The deep learning model efficiently captured signatures of purifying selection, consequently elucidating the interaction between positive and negative selection in deeply sequenced blood samples.

The utility of deep learning also extends to the areas of image processing and feature selection for cancer prognosis. Shirazi et al. [87] devised a deep convolutional neural network for segmenting whole-slide pathology images in glioblastoma, revealing that spatially resolved gene signatures correlated strongly with survival in genetically defined patient groups. In contrast, Yin et al. [90] utilized a CNN model for survival prediction based on prognosis-related cascaded Wx feature selection, demonstrating its high predictive power.

Finally, studies such as those by Wang et al. [89] and Li et al. [91] incorporated innovative strategies in their deep learning applications. Wang et al. employed bidirectional long short-term memory to examine the microbial model organism Saccharomyces Cerevisiae, intending to infer pan-cancer-associated genes. Li et al. combined deep learning neural networks with MRI radiomics to construct an immunophenotype-associated mRNA signature for predicting overall survival in patients with lower-grade glioma. These diverse deep learning applications underline the field’s potential in furthering cancer prognosis.

## 9. Survival Analysis with Generic Deep Learning with Modifications

Deep learning, a subfield of artificial intelligence, has emerged as a powerful tool for survival analysis in cancer prognosis [116]. This advanced machine learning technique entails the use of multiple layers of artificial neural networks to uncover intricate patterns in complex data. The growth in the use of generic deep learning models in cancer prognosis can be attributed to their ability to accommodate high-dimensional data, handle nonlinear relationships, and identify intricate interactions between features.

The recent trend in survival analysis with deep learning involves the development of architectures that are not only robust but also interpretable. This means that these models are not only proficient at identifying patterns that can predict cancer survival outcomes, but they also provide insights into the underlying biological mechanisms, which can be translated into clinical practice.

Recent studies have been geared towards enhancing the performance and interpretability of these models. For instance, attention mechanisms have been integrated into deep learning architectures to identify important features for prediction. This not only improves the performance of the models but also provides insights into which features are crucial in predicting survival outcomes. Table 7 summarizes recent studies.

The landscape of survival analysis leveraging generic deep learning has been enriched with several innovative modifications. The models proposed by Hou et al. [92] and Lee et al. [93] were aimed at predicting patient prognosis, employing both histopathology risk scores and whole-genome data, respectively. Hou et al. [92] improved upon their predecessors by combining histopathological information and hub genes from mRNA data for hepatocellular carcinoma prognosis, achieving a high concordance index. Concurrently, the method by Lee et al. [93] focused on predicting cancer occurrence through the analysis of mutation types, presenting remarkable accuracy and specificity.

Similarly, Jha et al. [94], Zheng et al. [95], and Al-Fatlawi et al. [96] explored different facets of cancer diagnosis and prognosis. Jha et al. [94] identified transcriptomic features commonly shared across different cancer types using feed-forward neural networks, introducing a novel perspective on common cancer transcriptome signatures. Meanwhile, Zheng et al. and Al-Fatlawi et al. [95,96] focused on bladder and pancreatic cancer, respectively, demonstrating the ability of deep learning to distinguish between different disease states and providing substantial diagnostic accuracy.

The applications of deep learning in cancer prognosis have extended beyond traditional genomic data. Elsharawy et al. [97] developed an AI-based breast cancer grading model utilizing images from the TCGA. The model exhibited significant potential in gene discovery and second opinions. In a similar vein, Ye et al. [98] and Guo et al. [99] proposed models for predicting susceptibility to ovarian cancer and prognosis of prostate cancer, respectively, underlining the versatility of deep learning techniques across various cancer types.

A novel trend in survival analysis is the incorporation of network-based approaches. Ramirez et al. [100] introduced Surv_GCNN, a graph convolution neural network approach for cancer survival prediction, which outperformed traditional models in multiple cancer types. Chen et al. [101] identified immune subtypes in gastric cancer using deep learning on whole-slide images, providing further insight into the use of deep learning in immunotherapeutic strategies.

The exploitation of deep learning in disease staging and prognosis prediction has also seen considerable advancement. Park et al. [102] developed a deep learning method for diagnosing non-alcoholic fatty liver disease and its association with hepatocellular carcinoma, demonstrating reliable performance. Ma et al. [104] constructed a prognostic model for cervical cancer with considerable predictive power, highlighting the potential of AI algorithms in optimizing prognosis models.

Lastly, several studies have employed deep learning in association with genetic abnormalities and their implications in cancer prognosis. Del Carmen et al. [105] identified chromosomal region alterations associated with therapy response in rectal cancer, utilizing a deep-learning-based algorithm for disease-free survival and overall survival prediction. Huang et al. [106] explored the roles of immune microenvironment-related elements in hepatitis B virus-related diseases, while Yang et al. [107] investigated the role of ACSL4 in non-small cell lung cancer. The studies underline the ability of deep learning to reveal the intricate links between genetic aberrations and cancer prognosis, further enhancing our understanding of the disease’s complex mechanisms.

## 10. Conclusions

In our comprehensive exploration of the existing literature, we emphasized the significant role that deep learning methodologies have played in survival analysis of cancer using genomic data. These advanced computational techniques have greatly improved the predictive capabilities of survival analysis and have provided a more nuanced understanding of complex genomic data.

Autoencoders, for instance, have shown great potential in extracting low-dimensional features from high-dimensional genomic data, and transfer learning techniques have allowed the leveraging of pre-existing models, reducing computational resources and improving generalization on small datasets.

A notable trend we have observed is the increasing preference for multi-modal and multi-omics studies, indicating the versatility of deep learning in handling and integrating diverse data types to provide a more holistic view of cancer survival.

However, the field of deep learning in cancer survival analysis is still emerging. A significant challenge is the development of more interpretable and explainable models, overcoming the black box nature of current methodologies. We also highlighted the need for further exploration of advanced deep learning techniques, such as graph neural networks and transformers, in this context.

One specific area that holds promise is the application of contrastive learning with self-supervised learning for survival analysis, especially when there is a lack of labeled data. For instance, the genomic implications in laryngeal cancer, particularly concerning the invasion of the paraglottic space or interarytenoid space [117], could be analyzed with this methods.

This review gives an extensive overview of the state-of-the-art deep learning methodologies used in cancer prognosis. We believe the insights from our review will guide future research in this important field, leading to novel and groundbreaking discoveries.

## Figures and Tables

**Table 1:** Summary of deep learning methods in cancer prognosis studies.

Deep Learning Category	Brief Description
Autoencoders [31,32,33,34,35,36,37,38,39,40,41]	A specific type of artificial neural network architecture designed for unsupervised learning of efficient codings. Autoencoders learn to compress input data into a coded representation and then uncompress it back to the original input. This capability makes them especially useful for dimensionality reduction and feature extraction tasks, where they learn to preserve as much information as possible while representing data in a reduced-dimensional space.
Ensemble of deep learning methods/models [42,43,44,45,46,47,48,49]	An ensemble method combines predictions from several different deep learning models to improve the overall predictive accuracy. Each model in the ensemble contributes a portion of the overall prediction. Through the combination of diverse models, the ensemble method can exploit the strengths of each individual model while mitigating their weaknesses, leading to a more robust and accurate overall prediction.
Multi-modal/multi-omics deep learning models [50,51,52,53,54,55,56,57,58,59,60,61,62,63,64,65,66,67,68,69,70,71,72,73,74,75,76]	These are sophisticated techniques designed to integrate and analyze data from different sources or modalities (such as imaging, genomics, proteomics, etc.) to enhance learning performance. By integrating data from diverse sources, these models can capture complex and hidden patterns that may not be evident when each data type is analyzed separately.
Deep learning with transfer learning [77,78,79,80]	In the context of deep learning, transfer learning involves leveraging a pre-trained model (usually trained on a large-scale dataset) or transferring learned features from one task to another. The key advantage of transfer learning is that it can significantly improve learning efficiency and performance, especially when the target task has limited training data.
Deep learning for manifold representation [81,82,83,84,85,86,87,88,89,90,91]	These methods employ deep learning architectures to generate numerical vector representations or embeddings of features in a lower-dimensional space (a manifold). Such representations can be utilized to reveal and preserve the intrinsic structure and relationships within the data, which are instrumental for downstream tasks.
Deep learning (unspecified/generic) [92,93,94,95,96,97,98,99,100,101,102,103,104,105,106,107,108,109]	This category encompasses general deep learning approaches, which may include a variety of architectures and techniques. These approaches often involve several minor modifications or adaptations to cater to the specificities of the task at hand, without specializing in a particular method or model like the other categories.

**Table 2:** Contributions of recent studies utilizing autoencoders.

Author and Citation	Contributions
Chai et al. [31]	Developed a multi-omics data integration approach using a denoising autoencoder for robust cancer prognosis prediction.
Kirtania et al. [32]	Proposed DeepSGP, an autoencoder-based deep learning strategy for predicting glioblastoma multiforme (GBM) patient survival.
Franco et al. [33]	Evaluated the performance of four autoencoders for cancer subtype detection using multi-omics data, with better results than standard data fusion techniques.
Song et al. [34]	Implemented an autoencoder-based model for prognosis prediction in colorectal cancer, demonstrating superior performance in identifying survival-related features.
Lai et al. [35]	Presented a disease network-based deep learning approach using an autoencoder model for characterizing melanoma.
Yang et al. [36]	Introduced deep subspace fusion clustering (DSFC), an autoencoder-based method for cancer subtype prediction from multi-omic data.
Zhang et al. [37]	Utilized an autoencoder-based deep learning framework to integrate multi-omics data for muscle-invasive bladder cancer subtyping.
Tian et al. [38]	Adopted an autoencoder-based approach to identify survival-sensitive subtypes of gliomas with high prognostic prediction ability.
Zhang et al. [39]	Proposed deep latent space fusion (DLSF), a deep learning model integrating multi-omics data for disease subtype identification.
Al Mamun et al. [40]	Employed the concrete autoencoder (CAE) to identify prognostic long non-coding RNAs (lncRNAs) for different types of cancers.
Madhumita and Paul [41]	Proposed an autoencoder-assisted cancer subtyping framework for identifying clinically significant cancer subtypes.

**Table 3:** Contributions of recent studies utilizing ensemble methods.

Author and Citation	Contributions
Poirion et al. [42]	Introduced DeepProg, an ensemble of deep learning and machine learning models for prognosis prediction using multi-omics data.
Yang et al. [43]	Proposed FuzzyDeepCoxPH, a fusion of deep learning algorithms and fuzzy logic system for identifying high-risk missense mutation variants and genes related to cancer mortality.
Arya and Saha [44]	Proposed a bi-phase model that enhances prognosis prediction for breast cancer patients by combining gated attentive deep learning models and random forest classifiers.
Zi et al. [45]	Proposed a DNN model for the prediction of survival risk in HCC patients, demonstrating high accuracy.
Kaur et al. [46]	Proposed a parallel Bayesian hyperparameter optimized Stacked ensemble (BSense) model for breast cancer survival prediction.
Tamilmani et al. [47]	Proposed an improved generative adversarial network optimized with a mayfly optimization algorithm for cancer miRNA biomarker classification.
Chen and Wei [48]	Proposed an improved survival prediction model using deep learning and self-supervised learning, demonstrating superior performance on cancer datasets from TCGA.
Carreras et al. [49]	Employed a multi-faceted algorithm to predict overall survival in MCL patients, identifying genes predictive of survival across multiple common cancers.

**Table 4:** Contributions of recent studies with transfer learning.

Author and Citation	Contributions
Chai et al. [77]	Proposed a transfer learning-based Cox proportional hazards network (TCAP) to integrate multi-omics data for predicting bladder cancer prognosis.
Johnson et al. [78]	Proposed diagnostic evidence gauge of single cells, a deep transfer learning framework, to transfer disease information from patients to cells.
Shi et al. [79]	Developed a deep learning model, based on the VGG19 architecture and transfer learning strategy, for prognosis prediction in colorectal cancer based on histopathologic features of the tumor microenvironment.
Meng et al. [80]	Introduced SAVAE-Cox, a novel framework that incorporates an attention mechanism and an adversarial transfer learning strategy for survival analysis of high-dimensional transcriptome data.

**Table 5:** Contributions of recent studies integrating multi-modal/multi-omics data.

Author and Citation	Contributions
Schulz et al. [50]	Developed a multimodal deep learning model for prognosis prediction in clear-cell renal cell carcinoma.
Chen et al. [51]	Proposed pathomic fusion, a strategy for fusing histopathology and genomic features for improved cancer diagnosis and prognosis.
Zhang et al. [52]	Proposed a deep tensor survival model integrating multi-omics cancer data to improve cancer survival outcome prediction.
Malik et al. [53]	Integrated multi-omics data using a neural network framework to predict survival and drug response in breast cancer patients.
Hassanzadeh et al. [54]	Presented an integrated deep belief network that analyzes RNA, miRNA, and methylation molecular data to predict cancer survival and provide risk stratification.
Zhang et al. [55]	Presented OmiEmbed, a multi-task deep learning framework for multi-omics data.
Wei et al. [56]	Proposed a deep learning-based approach leveraging multi-omics data for biochemical relapse prediction in prostate dancer patients.
Karabacak et al. [57]	Utilized a CNN-based deep learning model to stratify low-grade gliomas using a multiple-gene signature and MRI data.
Park et al. [58]	Constructed a multi-omics data-affinitive artificial intelligence algorithm based on the graph convolutional network to predict non-small-cell lung cancer.
Steyaert et al. [59]	Developed a deep learning framework for multimodal data fusion for prognosis prediction in brain tumors.
Chen et al. [60]	Integrated radiomic features with genomic data to improve the survival analysis for non-small cell lung cancer patients.
Choi and Lee [61]	Developed Multi-PEN, a deep learning model for prognosis estimation in low-grade glioma patients.
Zhou et al. [62]	Developed a deep learning model to classify Nottingham prognostic index score levels for breast cancer patients, leveraging multi-omics data.
Islam et al. [63]	Proposed a radiogenomic overall survival prediction approach for GBM, integrating gene expression data with radiomic features.
Schmelz et al. [64]	Conducted in-depth analyses combining transcriptomic and genomic profiling in neuroblastoma patients, reporting continuous clonal evolution.
Yang et al. [65]	Developed HISMD, an immune subtyping system for HNSCC using multi-omics data and deep learning techniques on whole slide images.
Hira et al. [66]	Developed multi-omics analysis model for ovarian cancer using variational autoencoders.
Calabrese et al. [67]	Evaluated an artificial intelligence method for predicting clinically relevant genetic biomarkers from preoperative MRI in patients with glioblastoma.
Pan et al. [68]	Developed i-Modern, an integrated multi-omics deep learning network method, to identify potential therapeutic targets in glioma.
Tan et al. [69]	Presented a multi-modal fusion framework (MultiCoFusion) based on multi-task correlation learning for survival analysis and cancer grade classification.
Zhang et al. [70]	Conducted multi-omics data analyses to predict the prognosis of serous ovarian cancer (SOC) patients with principal component transformation (PCT).
Sharma et al. [71]	Developed a deep learning-based integrative model for survival time prediction in patients with HNSCC.
Tang et al. [72]	Developed a wavelet-based deep learning model for prognosis formulation in pancreatic adenocarcinoma.
Leng et al. [73]	Benchmarked deep learning methodologies for fusing multi-omics data, suggesting moGAT as the best performer for classification tasks, and efmmdVAE, efVAE, and IfmmdVAE for clustering tasks.
Carmichael et al. [74]	Proposed an integrative, exploratory analysis framework that uses angle-based joint.
Huang et al. [75]	Developed a model based on bidirectional deep neural networks (BiDNNs) to integrate DNA methylation and mRNA expression data for HCC samples.
Rescigno et al. [103]	Focused on characterizing CDK12-mutated mCRPC using a combination of targeted next-generation and exome sequencing techniques and deep learning.
Hu et al. [76]	Proposed a deep neural network, GCS-Net, for predicting gastric cancer prognosis based on biological information pathways.

**Table 6:** Contributions of recent studies with manifold representation learning.

Author and Citation	Contributions
Zhang and Kiryu [81]	Developed MODEC, an unsupervised clustering method using manifold optimization and deep learning for identifying cancer subtypes.
Zhang et al. [82]	Developed the deep Bayesian perturbation Cox network (DBP) to effectively predict survival outcomes in cancer patients dealing with high-dimensional datasets.
Gupta et al. [83]	Developed continuous representation of codon switches (CRCS), a deep learning-based method for generating numerical vector representations of mutations with applications in detecting cancer-related somatic mutations and predicting patient survival.
Kim et al. [84]	Used a novel deep learning-based method to predict survival in oral cancer by analyzing tumor-infiltrating lymphocyte profiles.
Li et al. [85]	Developed CRCNet, a deep learning model for predicting survival outcome and the benefit of adjuvant chemotherapy in stage II/III colorectal cancer (CRC) patients.
Li et al. [86]	Employed deep learning to identify genetic mechanisms underlying immunosuppression in the survival of oral squamous cell carcinoma (OSCC) patients.
Shirazi et al. [87]	Developed a deep convolutional neural network (DCNN) for segmentation of whole-slide pathology images in glioblastoma to identify novel tumour cell–perivascular niche interactions associated with poor survival.
Skead et al. [88]	Conducted a deep learning and population genetics study on age-related clonal hematopoiesis (ARCH), demonstrating high accuracy in discriminating between evolutionary classes and captured signatures of purifying selection.
Wang et al. [89]	Utilized bidirectional long short-term memory (BiLSTM) to infer pan-cancer associated genes by examining the microbial model organism Saccharomyces Cerevisiae (Yeast) by homology matching.
Yin et al. [90]	Developed a convolutional neural network (CNN) model, named the CNN-Cox model, for survival prediction based on prognosis-related cascaded Wx feature selection.
Li et al. [91]	Constructed an immunophenotype-associated mRNA signature (IMriskScore) for predicting overall survival in patients with lower-grade glioma using deep learning neural networks with MRI radiomics.

**Table 7:** Contributions of recent studies with generic deep learning with modifications.

Author and Citation	Contributions
Hou et al. [92]	Proposed an integrative histology-genomic analysis for HCC prognosis using deep learning, integrating histopathology risk scores and hub genes.
Lee et al. [93]	Proposed a deep learning model for predicting cancer occurrence by utilizing whole-genome data, demonstrating exceptional performance on the TCGA dataset.
Jha et al. [94]	Utilized neural networks to identify transcriptomic features shared across different cancer types, discovering common cancer transcriptome signatures.
Zheng et al. [95]	Developed deep learning-based models for accurate diagnosis and survival prediction in bladder cancer using histological images.
Al-Fatlawi et al. [96]	Utilized deep learning models to improve the diagnosis of pancreatic cancer using RNA-based variants from blood samples.
Elsharawy et al. [97]	Demonstrated the potential of an AI-based breast cancer grading model, trained using CNN on images from TCGA.
Ye et al. [98]	Proposed a deep learning-based method to predict genes susceptible to ovarian cancer, using a graph attention network (GAT) and a deep neural network (DNN).
Guo et al. [99]	Proposed a deep learning-based model, DLFscore, for the prognosis prediction and potential chemotherapy sensitivity in prostate cancer.
Ramirez et al. [100]	Introduced Surv_GCNN, a novel GCNN approach for cancer survival rate prediction, outperforming other models in multiple cancer types.
Chen et al. [101]	Identified immune subtypes and landscape of gastric cancer using a deep learning model trained on whole-slide images.
Park et al. [102]	Developed a deep learning method to diagnose different stages in NAFLD and its relationship with HCC.
Ma et al. [104]	Optimized the prognostic model of cervical cancer using AI and data mining technology, identifying DMCs and constructing a prognostic model.
Del Carmen et al. [105]	Studied the relationship between genetic lesions and response to neoadjuvant radiochemotherapy in locally advanced rectal cancer, identifying a genetic signature predicting response to treatment.
Huang et al. [106]	Explored the roles of immune microenvironment-related factors in hepatitis B virus-related diseases using AI-based model.
Yang et al. [107]	Investigated the role of ACSL4 in NSCLC and its link to the ferroptosis process using deep learning.
Mehmood et al. [108]	Employed deep learning to identify compounds potentially possessing superior affinity for KRAS mutants in colorectal cancer.
Wang et al. [109]	Proposed a method to predict long-term survival in lung cancer patients using gene expression data and a CNN-based deep learning model.

## Data Availability

No new data were created or analyzed in this study.
